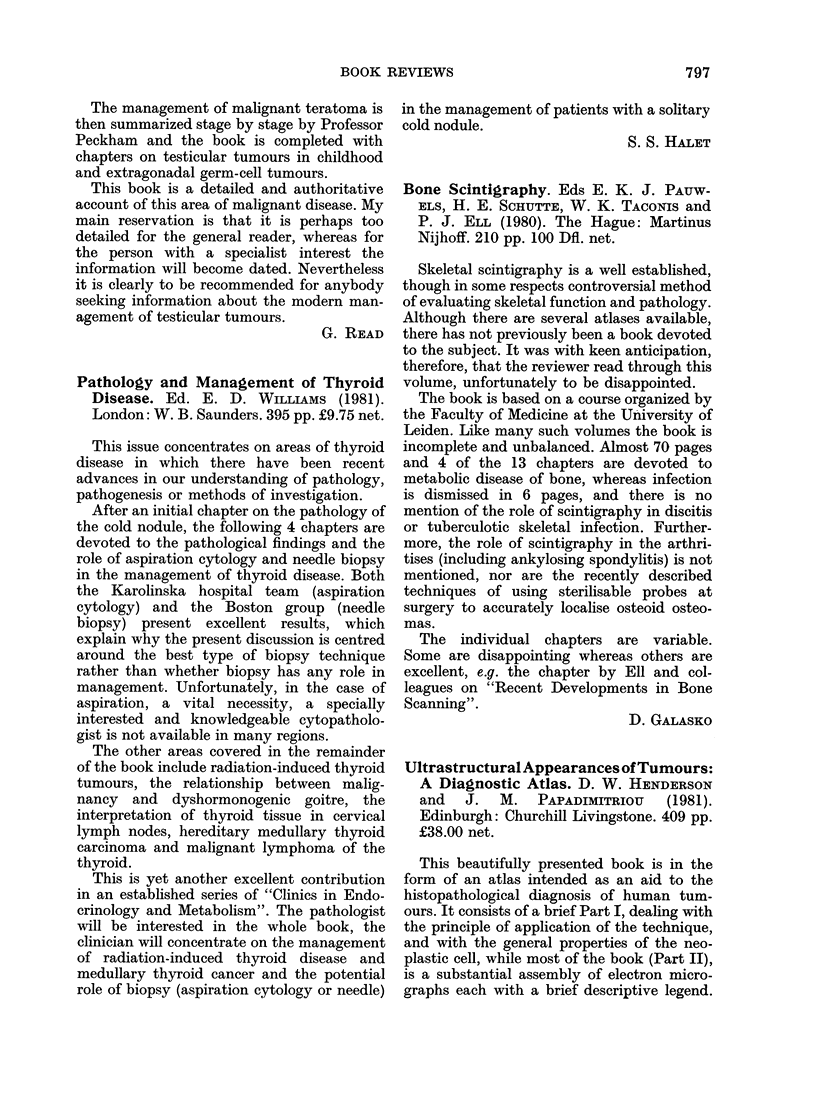# Pathology and Management of Thyroid Disease

**Published:** 1982-05

**Authors:** S. S. Halet


**Pathology and Management of Thyroid Disease.** Ed. E. D. WILLIAMS (1981). London: W. B. Saunders. 395 pp. £9.75 net.

This issue concentrates on areas of thyroid disease in which there have been recent advances in our understanding of pathology, pathogenesis or methods of investigation.

After an initial chapter on the pathology of the cold nodule, the following 4 chapters are devoted to the pathological findings and the role of aspiration cytology and needle biopsy in the management of thyroid disease. Both the Karolinska hospital team (aspiration cytology) and the Boston group (needle biopsy) present excellent results, which explain why the present discussion is centred around the best type of biopsy technique rather than whether biopsy has any role in management. Unfortunately, in the case of aspiration, a vital necessity, a specially interested and knowledgeable cytopathologist is not available in many regions.

The other areas covered in the remainder of the book include radiation-induced thyroid tumours, the relationship between malignancy and dyshormonogenic goitre, the interpretation of thyroid tissue in cervical lymph nodes, hereditary medullary thyroid carcinoma and malignant lymphoma of the thyroid.

This is yet another excellent contribution in an established series of "Clinics in Endocrinology and Metabolism". The pathologist will be interested in the whole book, the clinician will concentrate on the management of radiation-induced thyroid disease and medullary thyroid cancer and the potential role of biopsy (aspiration cytology or needle) in the management of patients with a solitary cold nodule.

S. S. HALET